# Gamma-Band Auditory Steady-State Response and Attention: A Systemic Review

**DOI:** 10.3390/brainsci14090857

**Published:** 2024-08-26

**Authors:** Giedre Matulyte, Vykinta Parciauskaite, Jovana Bjekic, Evaldas Pipinis, Inga Griskova-Bulanova

**Affiliations:** 1 Life Sciences Centre, Institute of Biosciences, Vilnius University, Sauletekio ave 7, LT-10257 Vilnius, Lithuania; giedremat8@gmail.com (G.M.); vykinta.parciauskaite@gmail.com (V.P.); evaldas.pipinis@gmc.vu.lt (E.P.); 2 Human Neuroscience Group, Institute for Medical Research, University of Belgrade, Dr Subotića 4, 11000 Belgrade, Serbia; jovana.bjekic@imi.bg.ac.rs

**Keywords:** auditory steady-state response, ASSR, gamma-band, 40 Hz, attention

## Abstract

Auditory steady-state response (ASSR) is the result of the brain’s ability to follow and entrain its oscillatory activity to the phase and frequency of periodic auditory stimulation. Gamma-band ASSR has been increasingly investigated with intentions to apply it in neuropsychiatric disorders diagnosis as well as in brain–computer interface technologies. However, it is still debatable whether attention can influence ASSR, as the results of the attention effects of ASSR are equivocal. In our study, we aimed to systemically review all known articles related to the attentional modulation of gamma-band ASSRs. The initial literature search resulted in 1283 papers. After the removal of duplicates and ineligible articles, 49 original studies were included in the final analysis. Most analyzed studies demonstrated ASSR modulation with differing attention levels; however, studies providing mixed or non-significant results were also identified. The high versatility of methodological approaches including the utilized stimulus type and ASSR recording modality, as well as tasks employed to modulate attention, were detected and emphasized as the main causality of result inconsistencies across studies. Also, the impact of training, inter-individual variability, and time of focus was addressed.

## 1. Introduction

Auditory steady-state response (ASSR) is a brain response characterized by consistent frequency and phase over a certain period of time triggered by an auditory recurring stimulus [1]. The primary cortical source of ASSR has been attributed to the auditory cortex [2] with demonstrated contributions from other cortical areas [3], the brainstem [4] and thalamus [5].

In recent years, the vast majority of studies have utilized stimulation within the gamma frequency range (30–50 Hz) to elicit ASSR and measure the intrinsic ability of auditory neuronal ensembles to entrain with periodically presented stimuli in various neuropsychiatric conditions, including schizophrenia [6,7], mood disorders [8], autism [9], and attention deficit hyperactivity disorder [10]. In these populations, alterations and deficiency of gamma-range ASSRs have been demonstrated [6,7,8,9,10], which was interpreted as a reflection of altered cognitive processes [11] and changes in inhibition/excitation balance [12]. Indeed, studies found an association between gamma-range ASSRs and different cognitive processes including the basic speed of cognitive processing [13] ability to temporarily store and manipulate the information [14] all the way to the ability to solve complex reasoning tasks [15]. On the cellular level, gamma-range ASSRs are assumed to reflect the dynamic interplay between excitatory pyramidal cells and parvalbumin-positive interneurons and their reciprocal excitatory and inhibitory interactions—the processes that are sensitive to different neurochemical modulators including drug compounds such as dexamphetamine [16], Δ-9-tetrahydrocanabinol [17], psilocybin [18], and even natural steroid hormones [19].

Moreover, gamma-range ASSRs are known to be sensitive to state-related factors, such as arousal levels [20] or participants’ consciousness level [21,22]. Therefore, it has been suggested that ASSRs might also be influenced by a person’s momentary state of attention. Even more so, because selective attention modulates neural processing in auditory systems [23,24], and there is an abundance of cognitive research showing that attention modulates perception across different modalities at both the sensory and neurophysiological level [25,26]. By extension, understanding of attentional modulation of ASSRs is essential in the context of neuropsychiatric disorders, where the reliability and validity of ASSRs used as biomarkers are likely to be affected by patients’ attentional state. Finally, precise identification of attentional modulation of ASSRs is of particular concern for the development of neurotechnological applications such as neurofeedback of brain–computer interface systems.

Therefore, it is no surprise that the study of attentional modulation of ASSRs spans almost 40 years. The initial study on the assessment of attentional effects on ASSRs failed to demonstrate any effect [27]. However, further research provided both positive and negative findings regarding the sensitivity of ASSRs to attention [28,29,30] or distraction [31,32]. Nevertheless, to the best of our knowledge, there have been no attempts to systematically compile and review the existing body of evidence on the attentional neuromodulation of ASSRs.

Here, we aim to systematize the current state of knowledge and critically evaluate previous studies addressing the attentional modulation of gamma-range ASSRs, to enable a better understanding of the complex interplay between these phenomena and foster ASSR usage as an individual biomarker. Aside from providing an overview of the existing evidence, special emphasis is put on methodological aspects of the studies, to enable exposing gaps in knowledge and possible methodological sources of disparate findings.

## 2. Methods

### 2.1. Literature Search

**Figure 1:**
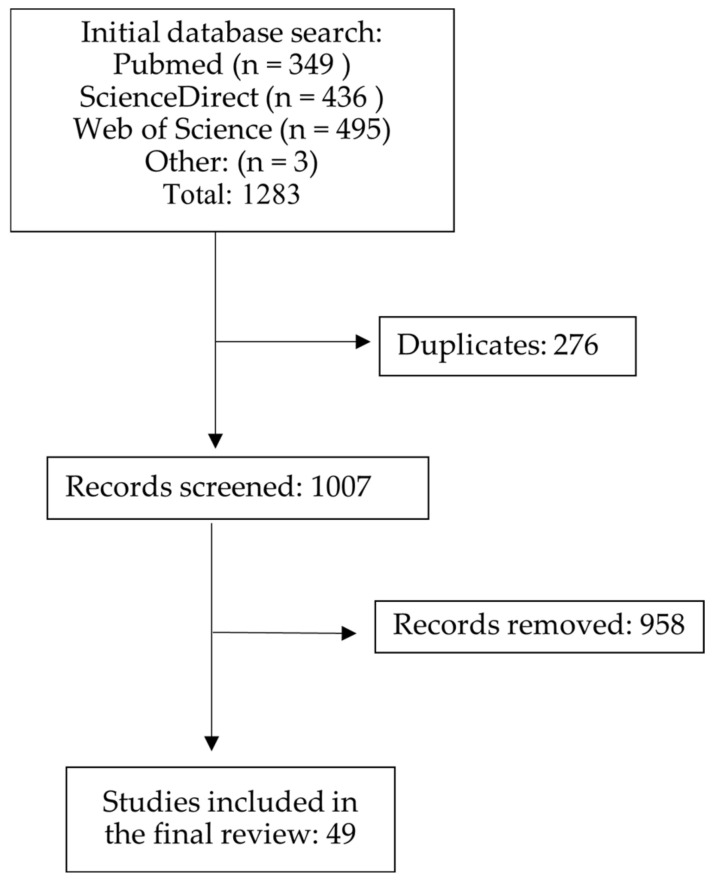
The schema of the search process and study selection.

Literature was collected using online searches in the PubMed, ScienceDirect, and Web of Science databases. The search was performed from September to November 2023. The following terms were used: “attention” AND (“auditory steady state response” OR “auditory entrainment” OR “envelope following”) for the ScienceDirect database, (“auditory” AND (“steady state” OR entrainment OR “envelope following”) AND “attention”) for PubMed, (“auditory” AND (“steady state” OR entrainment OR “envelope following”) AND “attention” for Web of Science. Rayyan [33] was used to remove any duplicates and select eligible studies from the database findings. Initially, the titles and abstracts were reviewed for selection criteria. If the information provided by the abstract was insufficient, the methodology section of the papers was analyzed. Irrelevant to the present review and papers in non-English were removed from further analysis. The flowchart of the selection procedure is presented in Figure 1.

### 2.2. Study Selection

For study selection, the following inclusion criteria were used: (1) original human studies in which the participants were ≥18 years old; (2) gamma-range (30–120 Hz) auditory stimulation was used; (3) EEG/MEG methods employed for recording responses and attention to auditory stimuli 4) a statistical comparison of ASSR measures in different attention conditions was reported. The exclusion criteria were set as follows: (1) animal studies; (2) studies measuring ASSRs in frequencies other than gamma-range (30–120 Hz); (3) attention to auditory stimulus was not experimentally manipulated; (4) studies in which ASSRs were collected during altered states (e.g., during sleep, anesthesia, or hallucinations); (5) studies in which ASSRs was modulated using brain-stimulation techniques (e.g., transcranial electrical or magnetic stimulation); (7) papers published in non-English languages; and (8) other types of publications such as conference reports, reviews, etc.

### 2.3. Data Extraction

The following information was extracted for each article (Table 1): (1) sample (type, size, age, and gender composition); (2) tasks/conditions that were used to modulate attention to the presented auditory stimuli; (3) auditory stimulation settings (stimulation frequencies, type, stimulus presentation technique); (4) the EEG/MEG assessment (measures, sites); and (5) effect of attention manipulation on ASSR measures. All studies have been assessed for the risk of bias (ROB2) by two independent researchers.

## 3. Results

The literature search resulted in a total of 1283 articles. After the exclusion of duplicates, 1007 papers remained. Articles that did not fulfill the inclusion requirements were removed from further analysis, leaving 49 studies, reporting 55 experiments, included in the final review. It is important to note that, in some papers, more than one experiment, stimulation type, or response recording mode was used, and they were presented separately (see Table 1). Overall, the reviewed studies showed a low risk of bias across all domains (see Supplementary Materials for each study risk-of-bias assessment, and the summary plot).

Healthy adult participants were involved in all the studies, while two studies also included participants with tinnitus [53,55] and two papers included patients with schizophrenia [35,41]. EEG was used as a recording technique in the majority of the studies (n = 35), while MEG was used in 13 studies, and only 1 study recorded both MEG and EEG [46]. Amplitude-modulated (AM) sounds (including speech modulation) were utilized most prevalently (n = 36). Nine articles employed click trains, while two studies used AM chirps [30,31], and one study utilized speech sounds [42]. Voicikas et al. [69] used both flutter AM tones and click trains. Binaural stimulation was utilized most (n = 33) while several studies employed monoaural or/and dichotic stimulation methods (n = 10). It is of note that the way in which auditory stimuli were presented was not explicitly stated in four papers. Only in two studies were auditory stimuli delivered through speakers [36,54] while all other studies delivered it using headphones.

The majority of studies evaluated ASSRs at 40 Hz or near 40 Hz. Three studies additionally evaluated responses at higher gamma-band (>80 Hz) [30,31,66], while three more studies focused solely on higher gamma-range ASSRs [42,44,68].

Power/amplitude (n = 43) and phase-locking index (n = 20) dominated as measures used to evaluate and compare ASSRs between different conditions. Several studies also evaluated the signal-to-noise ratio [44,54,61,73], latency [57,58,59], total field power [38,53,55], and dipole orientation [37,38], while single studies estimated total intensity [40], source strength [52] and global field synchronization [32].

Most of the EEG studies provided results for frontocentral channels (n = 24). In MEG studies, responses were mostly averaged across sensors covering all heads [42,48,49,50,73] or focusing on temporal lobe locations [29,34,46,51]. Notably, nine studies did not provide exact information on electrodes/sensors used for the evaluation of the responses [36,43,52,54,60,64,67,68,70].

Attentional modulation was performed by employing a variety of different tasks and conditions. Participants’ attention to the stimulation was manipulated by employing auditory detection/discrimination tasks (n = 26), while visual stimuli (n = 15) and tasks (n = 10) were used as a distraction. Numerous studies have utilized attention modulation tasks in the auditory modality (n = 22) only. Four studies have used ASSR to evaluate visual load [65,66,73,74]. In addition, passive listening (n = 7) and eyes closed/open [32,40,69] conditions were included in several works. Most frequently, the attend condition was compared to the distraction condition (active attention was required for another task, n = 32), to the unattended condition (auditory stimuli ignored, n = 13), or to passive listening (n = 10).

Looking at the outcomes, a vast majority of the studies showed attentional modulation of ASSRs (n = 39). The increment of the measures related to response strength and phase synchronization with attention to auditory stimulation was shown in more than half of the papers (n = 28), while reduced latency was observed in three [57,58,59]. A decrease in measures associated with distraction [31,32,40,61,73,74,75], masking noises [52], or attention shift between modalities [46] was found in nine papers. Meanwhile, Rockstroh et al. [56] and Weisz et al. [70] observed a decrement of measures with attention to target stimuli. No effects of attention on gamma-band ASSR were shown in 10 papers—3 of those addressing higher gamma (>80 Hz) ASSR [30,66,68]. In addition, three papers reported conflicting results: Voicikas et al. [69], showed no attention effects on ASSR evoked by flutter AM tones but found changes when ASSR was elicited by click train stimulation [69]; Keitel et al. [46] did not observe any changes for MEG recordings but detected expected changes for EEG data; and Gander et al. 2010 [39] reported effect of attention in one experiment, but did not observe it in the other. Importantly, 15 out of 17 mentions of negative findings did not show the effect of attention on ASSRs evoked with AM tones. Moreover, in 10 papers, insignificant effects were demonstrated using EEG.

## 4. Discussion

With increasing research and potential utilization of ASSRs in clinical practice and neurotechnological applications, attention has become frequently studied as an important factor that can influence ASSRs. However, over the years, studies exploring the effect of attentional demands on gamma-range ASSRs have provided somewhat inconsistent results, and no systematic attempts were made to generalize the known effects. The aim of this review was to summarize and critically assess the existing studies of attention effects on ASSR, from both methodological perspective and level of evidence. Over the years, starting with the study by Linden et al. [27], forty-nine original research papers reporting the results of 55 experiments were published. The vast majority of studies (n = 39) showed changes in gamma-range ASSR measures as a result of different attentional demands. Ten studies reported null effects, with three of those addressing high-frequency gamma activity. Additionally, in three papers, conflicting results were outlined depending on stimulation type, recording modality, or experiment. Importantly, the evidence presented here primarily refers to the attentional ASSR neuromodulation in healthy people, as only three studies included clinical samples with two demonstrating no effect in patients.

Of those studies showing attentional neuromodulation, the increase in ASSR measures with attention was observed in 28 reports, shorter ASSR latency was observed in 3 papers, and a reduction in ASSR measures with distraction—in 9 studies. The effect reported in two papers was opposite in direction to the expected change [56,70]. Thus, despite most studies showing attentional modulation of ASSR, conflicting reports are found in the literature. This is likely due to the identified high variability of methodological approaches, including both technical aspects of experiments (type of stimulation used, ASSR recording modality, and analysis settings), as well as tasks utilized to modulate attention, and, probably also, individual differences between studied participants. To address these issues in more detail, we will discuss the results focusing on the methods employed in the reviewed studies.

In the papers included in the review, both intramodal and intermodal attention were manipulated, and inconsistent results were shown with both approaches. To illustrate, no changes in ASSRs with shifts in both intramodal attention [39,41,47,51,68] and intermodal attention [27,30,54,65,66,68] were reported. Moreover, manipulations of visual load during intermodal attention experiments, including the detection of targets of different complexity [65,66], performance of reading and visual search tasks [40], playing different levels of the Tetris game [61], or engaging in a modified N-back task [73,74], mostly resulted in attenuation of ASSR measures with increasing task load. However, the changes appear to be conflicting, as no significant impact of visual load [65,66] or task difficulty [40] was also observed. In addition, Keitel et al. [46] demonstrated that a shift from one modality to the other in concurrent visual–auditory tasks decreased the response of the ignored modality, but directing attention to a specific modality did not result in ASSR changes. Finally, Griskova et al. [40] observed decreased ASSR with visual distraction tasks only compared to eyes closed (unfocused) conditions, and not to attention-demanding conditions [40]. All these suggest that certain aspects of the attention-modulating task and its performance could influence the outcome, and thus need to be taken into account in further studies.

To illustrate, Tsuruhara et al. [76] (a conference report not included in the review) demonstrated enhanced ASSRs in pilots gaining more experience on a flying task, which could be attributed both to attentional demands toward the main task and potentially to changes in neuronal plasticity due to repeated exposure to the periodic sounds. The repeated exposure to auditory stimuli was shown to decrease the phase delay between the 40 Hz response and stimulus waveforms [38,55]; the effect being observed 24–72 h after the first session, as well as after ten training sessions [38]. Moreover, Roberts et al. [55] demonstrated that effects can vary in healthy controls and patients with tinnitus: the authors observed no significant changes in phase but increased amplitudes with training in the tinnitus group, while no changes in amplitude but decreased phase delay were found in the healthy controls. The effects were attributed to the expression of neural plasticity [55]. Furthermore, the duration of “focused time” (attention focused on the stimulation) could affect the ASSR outcome, as demonstrated by Gander et al. [39]. Authors observed a significant ASSR increment when attention was needed for 1 s, but not when the focus time was 2 min. However, they observed significant ASSR enhancement when the 2 min trials were analyzed by dividing them into shorter 500 ms segments preceding responses to targets, suggesting that the effect of attention may be associated with the required concentration time.

The above-mentioned are particularly relevant in light of the increasing use of gamma-range ASSRs as potential biomarkers of psychosis [7]. Several attempts were made to compare attentional effects in healthy controls and patients with schizophrenia. Coffman et al. [35] showed enhancement of 40 Hz ASSR with attention only in healthy subjects but not in patients with schizophrenia when subjects were required to count stimuli and report every 7th in a row, thus supplementing the existing data on abnormal gamma-band ASSRs in patients [6,7,35], a finding that could be associated with the overall cognitive decline and altered attentional processes in particular [7]. However, Hamm et al. [41], failed to find significant effects of attention in both healthy subjects and schizophrenia patients when subjects were required to attend to auditory stimulation and detect unmodulated pure-tone targets among 40 Hz AM standards. Without follow-up studies, it is difficult to judge if this discrepancy is due to the specific characteristics of the samples or purely due to the lack of power issue stemming from the small sample size.

When ASSRs are recorded with EEG, the focal fronto-central response is observed [13] due to the configuration of sources generating it [2], and the effect of attention is most frequently assessed in these locations, where a response is clearly detected. However, several works observed a different spatial pattern of attentional modulation, pointing to the potential intricate interplay with other brain areas [34] that should be further addressed. For example, De Jong et al. [13] found enhanced ASSR amplitudes in the divided attention condition only in the occipital regions and failed to observe significant effects in fronto-central locations where ASSRs are most pronounced. Authors attributed this effect to the enhanced influence of auditory input on neural activity in the occipital cortex. In line with that, performing a motor action has been noted to perturb steady-state responses [56,77], suggesting a possible interference between auditory stimuli processing and the execution of a motor action that was required in the majority of the studies. Finally, although several works demonstrated gamma-range ASSR enhancement in the hemisphere contralateral to the attended auditory source [29,34,60,67], Weisz et al. [70] found decreased ASSRs in the right auditory cortex when participants were cued to focus on the stimuli presented to the right ear. Authors suggested that it reflects the default tendency of 40 Hz sounds to be processed by the right auditory cortex, which is actively suppressed when attention needs to be allocated to the right ear input, hinting that the effect of attention in complex stimulation settings might be even more complicated. Still, it is important to mention that 25% of the reviewed studies provided insufficient information on the localization of the evaluated signal (EEG or MEG sensors), thus preventing further generalization.

Importantly, as demonstrated in this review, the nature of the stimulus used to elicit ASSR is of importance. Non-significant findings were reported in experiments mostly using AM sounds. To illustrate, Voicikas et al. [69] demonstrated that ASSR elicited with clicks (brief bursts of white noise) increased when attention was paid to stimulation, while no effect was detected for ASSR in response to flutter amplitude-modulated tones (440 Hz carrier frequency). In line with that, a decrease in higher gamma response with distraction was observed for chirp stimuli with a high-frequency (1200 Hz) carrier [31], but no changes were seen for chirps with a low-frequency (440 Hz) carrier [30].

In conclusion, this review highlights the intricate interplay between attention and gamma-range ASSRs. A vast majority of the studies showed attentional modulation of ASSRs (n = 39) with varying attentional demands (the evidence primarily refers to the attentional ASSR neuromodulation in healthy samples)—with stronger and/or more synchronized responses obtained when attention is paid to stimulation, or weaker and/or less synchronized responses when attention is distracted from auditory stimulation. However, inconsistencies still arise due to methodological variations. The effects of attention across tasks and modalities are mixed, often exhibiting non-linear relationships and sometimes resulting in no significant effects. Factors such as training effects, attention duration, and inter-individual variability further complicate the understanding of the attention–ASSR relationship. The localization of ASSR signals and stimulus nature seem to be critical factors, as shown by differing effects with different recording modalities and stimulus types. Addressing these methodological challenges is crucial for advancing ASSRs’ clinical utility in conditions where attentional effects are difficult to control (i.e., schizophrenia, bipolar disorder, ADHD, autism) as there is currently insufficient evidence to reliably provide evidence-based support for the standardization of the assessment. Further research with larger samples and standardized methodologies is needed to fully understand attentional modulation’s mechanisms and its implications for clinical practice (i.e., the optimal instructions for data collection) and technology (i.e., the optimal experimental settings for best performance).

## Figures and Tables

**Table 1:** Characteristics of the studies included in the review.

Article	Sample Size (Age, Males)	Tasks/Conditions	Stimuli (Frequency; Type; Stimuli Presentation)	ASSR Measures and Site	Results
Albrecht et al., 2013 [16]	Healthy: 44 (19–48 years; 26 males)	Counting targets (20%, 20 Hz, or 40 Hz)	20/40 Hz click trains	EEG, 32 channels; Power and PLI (FCz)	PLI and power increased with attention for both 20 Hz and 40 Hz ASSR
Alegre et al., 2008 [31]	Healthy: 12 (27.6 years; 8 males)	(1) Attend to the sound; (2) Read a novel	1–120 Hz, 1200 Hz AM chirps; binaural stimuli presentation	EEG, 64 channels; PLI and power. (all channels, Fz)	Power decreased with distraction in the range of 80–120 Hz
Bharadwaj et al., 2014 [34]	Healthy: 10 (20–40 years; 8 males)	(1) Count the vowel (letter E); attention to the left or the right stream (signified with visual cues); (2) Ignore sounds and count the visual dot flickers	35 Hz and 45 Hz AM vowels in different streams; dichotic stimuli presentation	MEG, 306 channels; PLI (whole-brain) and power (20 strongest sources in each auditory ROI)	Power and PLI of 35 or 45 Hz ASSR increased with attention in contralateral auditory cortical areas
Coffman et al., 2022 [35]	Healthy: 32 (24.7 years; 22 males) Schizophrenia: 25 (23.3 years; 15 males)	(1) Count auditory stimuli; button press on every 7th stimulus; (2) Ignore the stimuli and watch a video	40 Hz click trains; binaural stimuli presentation	EEG, 61 channels; Evoked power and PLI (F1, Fz, F2, FC1, FCz, FC2)	Power and PLI of 40 Hz ASSR increased with attention in healthy but not in SZ patients
De Jong et al., 2010 [36]	Healthy: 10 (21.1 years; 3 males) (9 reported)	(1) Detect visual (increase or decrease in the main brightness of the 24 Hz flicker) or/and auditory (increase or decrease in the mean loudness of the 40 Hz AM tone) target. Target probability—50%/50%; (2) Discriminate auditory and visual targets, indicate direction of change in volume or brightness. Five attention conditions for each task: Focused attention—100% auditory or 100% visual; divided attention—20%/80%, 50%/50%, 80%/20% of auditory/visual; button press	40 Hz, 500 Hz AM through loudspeaker	EEG, 70 channels; Mean amplitude (4 frontocentral electrodes with maximum amplitude and O9, Iz O10)	No significant effects
Gander et al., 2007 [37]	Healthy: 63 (20.6 years; 18 males)	For 31 subjects: (1) Passive listening with video; (2) Detect targets (amplitude change > 400 ms; 50/50%) in S1/S2 pair; button press; For 17 subjects: Passive listening with video; For 15 subjects: (1) Passive listening with video; (2) Passive listening without video	40.96 Hz; 2000 Hz AM; binaural stimuli presentation	EEG, 128 channels; Amplitude, phase and dipole orientation, 3D dipole location (Fz)	Amplitude of 40 Hz ASSR increased with attention. No effects for phase, dipole orientation, and dipole 3D location
Gander et al., 2010a [38]	Exp 1: Healthy: 63 (20.6 years; 18 males)	For 31 subjects: (1) Detect targets (amplitude change; one of the 78 AM pulses was the target); button press; (2) Passive listening with video; (3) Passive listening without video; For 17 subjects: Passive listening without video; For 15 subjects: Passive listening with video	40.96 Hz, 2000 Hz AM; binaural stimuli presentation	EEG; 128 channels; Amplitude, phase, dipole moments (Fz), 3D location, total field power (all channels)	Amplitude of 40 Hz ASSR increased with attention. No effect for phase, dipole orientation, and 3D location
	Exp 2: Healthy: 18 (21.9 years; 8 males)	(1) Detect targets (amplitude change; 2/3 of stimuli contained a single amplitude enhanced 40 Hz pulse); button press; (2) Passive listening	40.96 Hz; 2000 Hz AM; binaural stimuli presentation	EEG; 128 channels; Amplitude and phase, dipole orientation, 3D dipole location (Fz)	Amplitude of 40 Hz ASSR increased with attention. No effect for phase, dipole orientation, and 3D location
Gander et al., 2010b [39]	Exp 1: Healthy: 34 (21.2 years; 14 males); divided into groups: 21 subjects in ‘1 s’ group, 13 in ‘2 min’ group	Simultaneous visual (16 Hz) and auditory streams. (1) Response to visual target (increase in the intensity); mouse click/button press; (2) Response to auditory target (increase in the intensity); mouse click/button press; (3) Passive condition (maintain focus on the fixation cross)	40.96 Hz, 2000 Hz AM; binaural stimuli presentation	EEG, 128 channels; Amplitude, phase, dipole power (FCz)	Amplitude and dipole power of 40 Hz ASSR increased when attention was required for 1 s to corresponding modality but not for 2 min intervals. No effects for phase
	Exp 2: Healthy: 39 (22.0 years; 17 males); divided into groups: 15 subjects in ‘1 s’ group, 24 in ‘2 min’ group	Simultaneous auditory streams. (1) Detect targets (increase in the amplitude); mouse click/button press; (2) Passive condition (maintain focus on the fixation cross)	40.96 Hz and 36.57 Hz simultaneously, 250 and 4100 Hz AM; binaural stimuli presentation		No significant effects
Griskova-Bulanova et al., 2011 [40]	Healthy: 11 (22.8 years; 6 males)	(1) Count 20 Hz stimuli, (2) Count 40 Hz stimuli; (3) Eyes closed; (4) Eyes open; (5) Read; (6) Visual search-task	20/40 Hz click trains; binaural stimuli presentation	EEG, 32 channels;PLI, evoked amplitude, total intensity (F3, Fz, F4, C3, Cz, C4, P3, Pz and P4)	PLI and amplitude of 40 Hz ASSR decreased with distraction tasks compared to closed eyes condition. No effect for total intensity of 40 Hz ASSR
Griskova-Bulanova et al., 2018 [32]	Healthy: 27 (23.2 years; 27 males)	(1) Count stimuli; (2) Read; (3) Eyes closed	40 Hz click trains; binaural stimuli presentation	EEG; 64 channels; Global field synchronization (all channels)	GFS of 40 Hz ASSR increased with attention and closed eyes. GFS of 40 Hz ASSR decreased with distraction
Hamm et al., 2015 [41]	Healthy: 18 (40.8 years; 11 males); Schizophrenia: 18 (45.6 years; 9 males)	(1) Detect targets (10%, unmodulated tones); button press; (2) Passive listening	40 Hz, 500/1000/2000 Hz AM;binaural stimuli presentation	EEG; 211 channels; Power (all channels)	No significant effects
Hartmann et al., 2019 [42]	Healthy: 38 (mean 24.4 years; 19 males) (34 reported, 24.4 years; 15 males)	Detect deviant auditory or visual stimuli; button press	114 Hz, /da/sound;	MEG, 306 channels); Power (102 magnetometers)	Power of 114 Hz ASSR increased with attention to the auditory domain
Herdman 2011 [43]	Healthy: 13 adults (22 years; 6 males) (10 reported, 5 males)	Attend to relevant deviant (175 ms)/standard (500 ms) 1200 Hz AM tones and detect targets (10%, 1200 Hz, 175 ms); button press. Ignore irrelevant deviant (175 ms)/standard (500 ms) 800 Hz AM	40 Hz, 800/1200 Hz AM; binaural stimuli presentation	MEG; 151 channels; Amplitude	Amplitude of 40 Hz ASSR increased with attention
Holmes et al., 2017 [44]	Healthy: 30 (24 reported, 20.5 years; 12 males)	(1) Detect deviant stimulus within the attended stream (low-, high-frequency); button press; (2) Attend to visual stimuli and ignore auditory	93/99/109 Hz, 1027/1343/2913 Hz AM; binaural stimuli presentation	EEG (Cz); EFR phase coherence, SNR, amplitude	Amplitude, phase coherence, and SNR of 93 Hz and 109 Hz ASSR increased with attention to tone stream
Keitel et al., 2011 [45]	Healthy: 16 (13 reported, 24.6 years; 6 males)	Perform auditory or visual lexical decision task (words 50%); button press	40 Hz AM multi-speech babble; binaural stimuli presentation	EEG. 64 channels; Maximum amplitude (Fz, FCz, F1, F2, FC1, FC2)	Amplitude of 40 Hz ASSR increased with attention to the auditory stream
Keitel et al., 2013 [46]	Healthy: 18 (16 reported, 26 years; 10 males)	Perform auditory or visual lexical decision task (words 50%); button press	40 Hz AM multi-speech babble; binaural stimuli presentation	MEG and EEG, 306 and 60 channels; Amplitude (EEG—T7, T9, TP7, FT7, C5 T8, T10, TP8, FT8, C6; MEG—lateral temporal sites)	Amplitudes of 40 Hz ASSR decreased when attention was shifted from audition to vision for EEG data but not MEG
Lazzouni et al., 2010 [29]	Healthy: 15 (26 years; 7 males)	Detect targets (10%, 950 Hz AM); button press	39 Hz, 1000 Hz AM to the right ear; 41 Hz, 1000 Hz AM to the left ear; monoaural/dichotic stimuli presentation	MEG, 275 channels; Amplitude (left/right temporal areas)	Amplitude of 40 Hz ASSR increased in the right hemisphere with attention (at the onset of carrier change during dichotic stimuli presentation)
Linden et al., 1987 [27]	Exp 1 Healthy: 8 (27–40 years; 6 males)	(1) Read; (2) Count targets (intensity increments)	40 Hz; 500 Hz AM; monoaural stimuli presentation	EEG, Cz; Amplitude, phase	No significant effects
	Exp 2 Healthy: 10 (22–38 years; 5 males)	(1) Read; (2) Count targets (AM change from 500 Hz to 535 Hz, and from 1000 Hz to 1050 Hz)	37 Hz to one ear, 41 Hz to the other; 500/1000 Hz AM; dichotic stimuli presentation	EEG; Fz, Cz, Pz; Amplitude, phase	No significant effects
Mahajan et al., 2014 [47]	Healthy: 23 (22–35 years; 13 males)	Detect target in a cued ear (15% congruent, 15% incongruent; for 16/23.5 Hz changed to 40 Hz; for 32.5/40 Hz changed to 12.5 Hz); button press	16/23.5 Hz and 32.5/40 Hz; white noise AM; dichotic stimuli presentation	EEG, 64 channels; Power (T7, T8)	No significant effects for 32.5/40 ASSR
Manting et al., 2020 [48]	Healthy: 29 (27 reported, 28.6 years; 18 males)	Listen to three melody streams of different pitches, attend to lowest (39 Hz) or highest (43 Hz); report the latest pitch direction; button press	43 Hz, 196–329 Hz AM; 41 Hz, 147–294 Hz AM; 39 Hz, 131–220 Hz AM; binaural stimuli presentation	MEG; 306 channels; Power; ERF amplitude (all gradiometer sensors)	Power and ERF amplitude of 39 Hz and 43 Hz increased with attention to corresponding frequency
Manting et al., 2021 [49]	Healthy: 29 (28.6 years; 20 males) (27 reported)	Listen to three melody streams of different pitches, attend to lowest (39 Hz) or highest (43 Hz); report the latest pitch direction; button press	43 Hz AM 329–523 Hz; 41 Hz AM 175–349 Hz; 39 Hz AM, 131–220 Hz; binaural stimuli presentation	MEG; 306 channels; Power (all gradiometer sensors)	Power of 39 Hz and 43 Hz ASSR increased with attention to corresponding frequency when the stimulus was present
Manting et al., 2022 [50]	Healthy: 28 (28.6 years; 19 males) (25 reported)	Listen to two overlapping melody streams of different pitches, attend to low (39 Hz) or high (43 Hz); report the latest pitch direction; button press	43 Hz, 329–523 Hz AM; 39 Hz, 131–220 Hz AM; binaural stimuli presentation	MEG; 306 channels; Power (all sensors)	Power of 39 Hz and 43 Hz increased with attention to corresponding frequency
Müller et al., 2009 [51]	Healthy:15 (25 years; 9 males) (13 reported)	Detect target in a cued ear (10%, amplitude modulation changes to 12.5/25 Hz); button press	20 Hz to one ear and 45 Hz to another ear 655 Hz AM; dichotic stimuli presentation	MEG, 148 channels; Power (left/right temporal sources)	No significant effects for 45 Hz ASSR
Okamoto et al., 2011 [52]	Healthy: 16 (26.2 years; 8 males)	(1) Detect auditory targets (10%, shift in carrier frequency) presented simultaneously with 8000 Hz white noise of different power (2) Ignore auditory stimulation presented simultaneously with 8000 Hz white noise of different power and detect visual targets; button press	40 Hz, 1000 Hz AM; binaural stimuli presentation	MEG, 275 channels; Source strength	Source strength of 40 Hz ASSR increased with attention and decreased with loud masking noises
Paul et al., 2014 [53]	Healthy/tinnitus: 30/30 Healthy: (16 reported, 64 years; 5 males and 11 reported, 53.9 years, 8 males); Tinnitus: 17 reported, 62.0 years; 10 males and 11 reported, 48.6 years, 7 males)	(1) Detect targets (~50%, amplitude-enhanced pulse); button press; (2) Passive listening (ignore stimulation)	40.96 Hz, 500 and 5000 Hz AM binaural stimuli presentation	EEG; 128 channels; Total field power (all electrodes)	Total field power of 40 Hz ASSR increased with attention
Pipinis et al., 2018 [30]	Healthy: 20 (21.8 years; 20 males)	(1) Read; (2) Count stimuli	1–120 Hz/120–1 Hz, 440 Hz AM chirps; binaural stimuli presentation	EEG, 64 channels; PLI and evoked amplitude (Fz, FCz, Cz)	No significant effects
Riels et al., 2020 [54]	Healthy 30 (mean 19 years; 7 males)	Exp 1. Detect transient tone amplitude reduction during rapid serial visual presentation of emotional images; button press	40.8 Hz, 600 Hz AM; through speakers	EEG; 129 channels; Amplitude, SNR (12 central and frontal channels)	No significant effects
		Exp 2. Passive viewing and listening task with anticipation of aversive white noise burst			No significant effects
Roberts et al., 2012 [55]	Healthy/tinnitus: 12/12 (Healthy: 11 reported, 53.9 years; 6 males; Tinnitus: 11 reported, 48.6 years; 7 males)	(1) Detect targets (66%, pulse of increased amplitude); button press (2) Passive listening	40.96 Hz; 5000 Hz AM; binaural stimuli presentation	EEG; 128 channels; Total field power, phase, amplitude (all channels)	Amplitude of 40 Hz ASSR increased with attention. No effect on phase
Rockstroh et al., 1996 [56]	Exp 1 Healthy: 45 (22.2 years; 45 males) (37 reported)	Detect targets (shift to 500 Hz AM or 2000 Hz AM, 30%); button press	40 Hz; 1000 Hz AM; monoaural stimuli presentation to the right ear	EEG; Fz, Cz, Pz; Amplitude	Amplitudes of 40 Hz ASSR decreased after frequency shift; at 350–400 ms more after shift to target. Amplitude recovery more pronounced after shift to standard
	Exp 2 Healthy: 10 (26.3 years; 5 males) (8 reported)	(1) Detect targets (shift to 500 Hz AM, 30%, for two subjects target was 2000 Hz AM in further sessions) button press; (2) Count targets	41 Hz; 1000 Hz AM; monoaural stimuli presentation to the right ear		Amplitudes of 40 Hz ASSR decreased to targets with active response; no effect with passive counting
Rohrbaugh et al., 1989 [57]	Healthy: 6 (27 years; 1 male)	(1) Count targets: easy (±200 Hz)/hard (±20 Hz); (2) Passive listening (80 tone bursts of 400 Hz or 600 Hz)	Background: 41 Hz pips, 1000 Hz AM; Foreground: 400 to 600 Hz tone bursts; binaural stimuli presentation	EEG; ASSR recording site −2 cm anterior from Cz; ERP (Fz, Cz, Pz); Peak-by-peak latency, amplitude	Latency reduction after the foreground stimulus in hard condition compared to passive. No effects for amplitude
Rohrbaugh et al., 1990a [58]	Healthy: 4 (23–28 years; 3 males)	(1) Count auditory targets: easy (±200 Hz)/difficult (±20 Hz); (2) Count visual targets: easy (standard circle and elongated 20° ellipse discrimination)/difficult (standard circle and elongated 60° ellipse discrimination)	Background: 39/41/45 Hz, pips, 1000 Hz AM; Foreground: 400 to 600 Hz tone bursts; binaural stimuli presentation	EEG; ASSR recording site −2 cm anterior from Cz; ERP from (Fz, Cz, Pz); Latency, peak-to-peak amplitude	Latencies and amplitude decreased within 200–300 ms after the auditory foreground stimulus
Rohrbaugh et al., 1990b [59]	Healthy: 4 (21–27 years; 2 males)	Count targets (30%): loud tone/soft tone	Background: 41 Hz pips, 1000 Hz AM; Foreground: 400 Hz tone bursts; binaural stimuli presentation	EEG; 2 cm anterior from Cz; Latency, peak-to-peak amplitude	Latency reduction after the foreground stimulus. No effect for amplitude
Ross et al., 2004 [60]	Healthy: 12 (23–54 years; 7 males)	(1) Discriminate target (10%, 30 Hz), button press; (2) Count visual stimuli, ignore sound	40 Hz, 500 Hz AM; monoaural stimuli presentation to the right ear	MEG; 151 channels; Dipole moment amplitude	Amplitudes of 40 Hz ASSR increased with attention, mostly in the left hemisphere
Roth et al., 2013 [61]	Healthy: 9 (21–40 years; 9 males) (8 reported)	(1) Play Tetris (easy/difficult levels); (2) Fixate on Tetris (keyboard disabled) while attending to the auditory stimuli (subtle variations in amplitude)	40 Hz; click trains; binaural stimuli presentation	EEG; 8 channels (F3, Fz, F4, Cz, P3, P4, O1, O2); Amplitude; SNR (channel with largest SNR)	SNR of 40 Hz ASSR increased with attention, and decreased with visuospatial task difficulty
Saupe et al., 2009a [62]	Healthy: 15 (7 males) (12 reported, 27.2 years; 7 males)	(1) Detect auditory target (30 Hz; 5 targets and 43 standards); button press; (2) Detect visual target (fixation cross change; 13 times); button press	40 Hz, 500 Hz AM; binaural stimuli presentation	EEG, 32 channels; Amplitude (Fz, FC1, FC2)	Amplitudes of 40 Hz ASSR increased with attention
Saupe et al., 2009b [63]	Healthy: 17 (9 males) (14 reported, 25.5 years, 8 males)	(1) Detect auditory target (30 Hz; 50%); (2) Detect visual target (letter, 50%)	40 Hz, 500 Hz AM; binaural stimuli presentation	EEG, 64 channels; Amplitude (two adjacent frontocentral electrodes with the largest amplitude)	Amplitudes of 40 Hz ASSR increased with attention
Saupe et al., 2013 [64]	Healthy: 15 (19–30 years; 23.5 years; 5 males)	(1) Active listening (press a button when stimulus onset asynchronies were <1.8 s or >5 s long); (2) Passive listening; (3) Self-generation (press a button to start stimuli); (4) Motor-control (no tone generated by button press)	40 Hz, 500 Hz AM; binaural stimuli presentation	EEG, 61 channels; Amplitude (Fz, FCz, F1, F2, FC1, FC2)	Amplitudes of 40 Hz ASSR increased with attention
Skosnik et al., 2007 [28]	Healthy: 15 (n/a; 5 males)	Count targets (20%, 20/40 Hz, counterbalanced)	20/40 Hz; click trains, binaural stimuli presentation	EEG, 12 channels; Power (F7, F8, Fz, C3, C4, Cz), PLF (Cz)	Power and PLF to 40 Hz increased with attention
Szychowska and Wiens 2020a [65]	Exp 1. Healthy: 43 (25.7 years; 20 males)	Respond to visual features of a cross (targets 20%): (1) low load—respond to red cross; (2) high load—respond to upright yellow and inverted green crosses; button press	40.96 Hz, 500 Hz AM; binaural stimuli presentation	EEG, 6 channels; Amplitude and PLI (Fz, FCz)	No significant effects
	Exp 2. Healthy: 45 (27.2 years; 21 males)	Respond to visual features of the letters (48 targets of 360) while ignoring the tone: (1) no-load (passive viewing); (2) low-load (color); (3) high load (color-name combinations); (4) very high load (combinations of name, color, and capitalization); button press	40.96 Hz, 500 Hz AM;binaural stimuli presentation		No significant effects
Szychowska and Wiens 2020b [66]	Healthy: 33 (27.09 years; 13 male)	Respond to visual features of the letters (48 targets of 247) while ignoring the tone: (1) no-load (passive viewing); (2) low-load (color); (3) high load (color-name combinations); button press	20.48/40.96/81.92 Hz, 500 Hz AM; binaural stimuli presentation	EEG; 6 channels; Amplitude and PLI (Fz, FCz)	No significant effects
Tanaka et al., 2021 [67]	Healthy: 18 (22.9 years; 18 male)	(1) Write down heard AM two-syllable words: to left, right, or both ears; (2) passive listening and watching a silent movie	35 and 45 Hz AM two-syllable words; diotic/dichotic stimuli presentation	MEG; 204 channels; Amplitude	Amplitude of 35 Hz and 45 Hz ASSRs increased with attention
Varghese et al., 2017 [68]	Exp 1. Healthy: 10 (18–28 years; 4 males) (9 reported)	Listen to streams of spoken digits and respond whenever two consecutive, increasing digits were heard in the attended ear (1) during monaural presentation; (2) while ignoring digits presented to the other ear in a dichotic listening; button press	97/113 Hz, click trains (vocoding) monaural/dichotic stimuli presentation	EEG, 32 channels; PLV (20 channels = 14 channels in common among all subjects + 6 random for each)	No significant effects
	Exp 2. Healthy: 13 (20–29 years; 3 males) (12 reported)	Attend to (1) a monaural digit stream; (2) to one stream in a dichotic listening; (3) to a visual digit stream during a monaural presentation; button press	97/113 Hz; click trains (vocoding); monaural/dichotic stimuli presentation		No significant effects
Voicikas et al., 2016 [69]	Healthy: 22 (22.6 years; 22 males)	(1) Count stimuli; (2) Eyes closed; (3) Read	40 Hz, 440 Hz AM, and click trains, binaural stimuli presentation	EEG, 64 channels; PLI, evoked amplitude (Fz, Cz)	PLI, peak PLI, and peak EA of 40 Hz ASSR elicited by click trains increased with attention versus distraction. No effects on ASSR evoked by AM
Weisz et al., 2012 [70]	Healthy: 11 (24–38 years; 5 males)	Define which ear stimulus is presented after the cue: informative (75%) or uninformative (50%). Indicate the side on which the target was perceived	19/42 Hz; 500/1300 or 1300/500 Hz AM; dichotic stimuli presentation	MEG; 275 channels; Power	Power of 42 Hz ASSR decreased in the right primary auditory cortex with the cue to focus on the right ear (target presented to the ipsilateral ear)
Wittekindt et al., 2014 [71]	Healthy: 23 (20–39 years; 10 males)	Detect target in cued stream (visual angle or auditory intensity change); button press	40 Hz; AM tones: f1 and f2 1000–2000 Hz, f2/f1 ratio 1.21; binaural stimuli presentation	EEG; 41 channels; Power (all channels)	Power of 40 Hz ASSR increased with attention
Yagura et al., 2021 [72]	Healthy: 22 (0 males); translators 7 experts (56.71 years) and 15 beginners (51.2 years)	(1) Simultaneous translation from Japanese to English; (2) Shadowing Japanese	40 Hz, click trains and speech sounds	EEG, 29 channels; PLI (F3, Fz, F4, C3, Cz, C4, P3, Pz P4)	PLI of 40 Hz ASSR increased in experts during the translation condition compared to the shadowing condition
Yokota and Naruse 2015 [73]	Healthy: 16 (20–23 years; 8 males).	Visual N-back task: 3 levels of difficulty, and no-load	40 Hz click trains	MEG, 148 channels; Power and PLI, SNR (all channels)	Power and PLI of 40 Hz ASSR decreased with increased task difficulty
Yokota et al., 2017 [74]	Healthy: 15 (20–35 years; 7 males).	Visual N-back task: 3 levels of difficulty, and no-load; walking on a treadmill	40 Hz, 500 Hz AM; binaural stimuli presentation	EEG, 8 channels; PLI (Fpz, FC3, FCz, FC4, O1, Oz, O2)	PLI of 40 Hz ASSR decreased with increased task difficulty
Zhang et al., 2018 [75]	Healthy: 15 (24 years; 9 males)	Detect targets (rising from 122 to 146 Hz; 100 stimuli): (1) in speech blocks target—vowels; (2) in non-speech—complex tones; both presented with or without a background noise (40 Hz AM); button press	40 Hz, 0.5–4 k Hz AM noise; binaural stimuli presentation	EEG, 60 channels; Amplitude, PLI (F3, FC3, C3, F4, FC4, C4))	PLI of 40 Hz ASSR decreased with speech and non-speech stimuli in both hemispheres, and amplitude after speech stimuli only on the left, after non-speech in both

AM—amplitude modulated, ASSR—auditory steady-state response, EEG—electroencephalography, ERF—event-related fields, ERP—event-related potential, GFS—global field synchronization, MEG—magnetoencephalography, PLI—phase-locking index, PLF—phase-locking factor, PLV—phase-locking value, ROI—region of interest, SNR—signal-to-noise ratio, SZ—schizophrenia.

## Supplementary Materials

The following supporting information can be downloaded at: https://www.mdpi.com/article/10.3390/brainsci14090857/s1, Figure S1: Risk-of-bias of individual studies; Figure S2: Risk-of-bias summary. Reference [78] is cited in the supplementary materials.
