# Supplementary material for: Gamma-Band Auditory Steady-State Response and Attention: A Systemic Review
Source: Brain Sci. 2024 Aug 26;14(9):857. doi: 10.3390/brainsci14090857 (PMC11430480; doi:10.3390/brainsci14090857)
Supplement: Supplementary file 1 [file brainsci-14-00857-s001.zip › brainsci-3089356-supplementary.pdf]

Supplementary Material

The visualization of the risk of bias was performed using Robvis tool (McGuinness & Higgins, 2020).

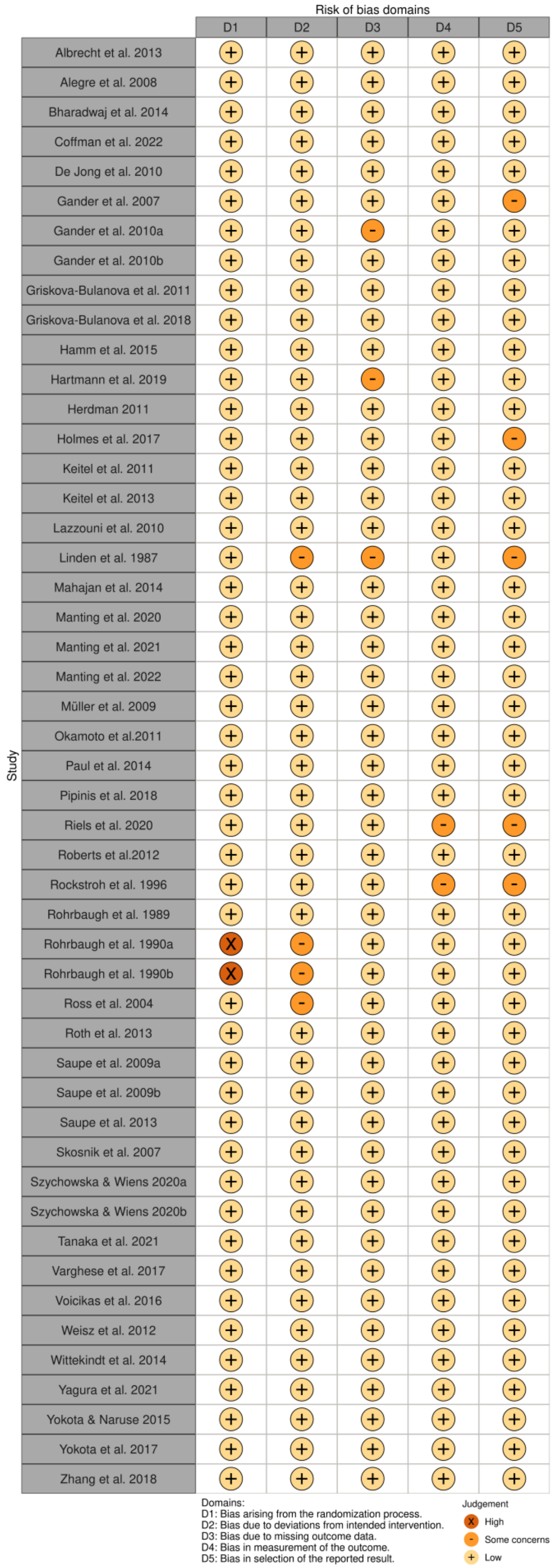

Figure S1. Risk-of-bias of individual studies.

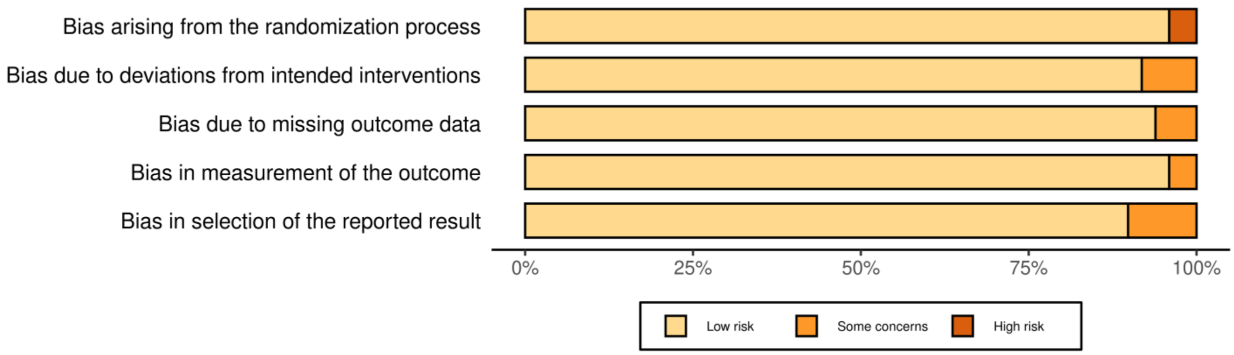

Figure S2. Risk-of-bias summary.

Reference

McGuinness, L.A.; Higgins, J.P.T. Risk-of-bias VISualization (robvis): An R package and Shiny web app for visualizing risk-of-bias assessments. *Res. Syn. Meth.* **2020**, *12*, 55–61. https://doi.org/10.1002/jrsm.1411.
